# Sp1 and COX2 expression is positively correlated with a poor prognosis in pancreatic ductal adenocarcinoma

**DOI:** 10.18632/oncotarget.8593

**Published:** 2016-04-05

**Authors:** Junjie Hang, Hai Hu, Junjie Huang, Ting Han, Meng Zhuo, Yangyang Zhou, Lei Wang, Yi Wang, Feng Jiao, Liwei Wang

**Affiliations:** ^1^ Department of Medical Oncology and Pancreatic Cancer Center, Shanghai General Hospital, Shanghai Jiao Tong University School of Medicine, Shanghai 201620, China; ^2^ Shanghai Key Laboratory of Pancreatic Disease, Shanghai 201620, China; ^3^ Department of Hematology, First Affiliated Hospital of Shenzhen University, Shenzhen 513000, China; ^4^ Department of Medical Oncology, First Affiliated Hospital of Bengbu Medical College, Anhui 233004, China; ^5^ Department of Radiation Oncology, Lianyungang First People's Hospital, Jiangsu 222002, China

**Keywords:** pancreatic ductal adenocarcinoma, specificity protein 1, cyclooxygenase-2, correlation, prognosis

## Abstract

Previous studies showed that celecoxib, a cyclooxygenase-2 (COX2) inhibitor, can inhibit angiogenesis and metastasis of pancreatic ductal adenocarcinoma (PDAC) via the suppression of specificity protein 1 (Sp1). In this study, we investigated the prognostic value of Sp1 and COX2 in 88 PDAC patients. Our study showed there was a positive correlation between Sp1 and COX2 expression (*P*=0.001) by using the Spearman's rank test. Pearson Chi-square test revealed that Sp1 and COX2 expression were positively associated with lymph node metastasis (*P*<0.05, both). In addition, the Kaplan–Meier analysis showed that patients with Sp1- or COX2-positive expression exhibited poorer overall survival (OS) than those with Sp1- or COX2-negative expression (*P*<0.05, all). Most importantly, Sp1- and COX2-negative patients had the best OS (*P*=0.01). In multivariate analysis, Sp1 expression (*P*=0.03), COX2 expression (*P*=0.04), and nuclear grade (*P*=0.009) were found to be independent predictors for OS. Moreover, we confirmed that Sp1 could upregulate the expression of COX2 in PDAC cell lines by western blot analysis, and both are of important prognostic value in PDAC.

## INTRODUCTION

Pancreatic ductal adenocarcinoma (PDAC) is the most common malignancy in the pancreas, with a resectable rate of only 20% and an overall 5-year survival rate of approximately 6% [1]. It was reported that around 330,000 patients died of PDAC during 2012, making it the seventh leading cause of cancer-related death worldwide [2]. Despite the improvement in diagnostic and therapeutic strategies, the prognosis for PDAC remains extremely dismal [3]. Hence, it is of vital importance to find more effective molecular prognostic markers for the treatment of PDAC patients in clinical practice [4, 5].

Specificity protein 1 (Sp1), a founding member of the Sp transcription factor family, is a sequence-specific and DNA-binding protein which affects cell proliferation, differentiation and apoptosis [6]. Accumulating evidence shows that the overexpression of Sp1 occurs frequently in a wide range of tumors [7, 8] and is correlated with unsatisfactory clinical outcome in these tumors [9–11]. Notably, previous studies revealed that Sp1 plays a critical role in the pathogenesis, aggressiveness and angiogenesis of PDAC [12–15]. Recently, evidence suggested that Sp1 may promote tumor growth by regulating the function of COX2, whose expression was also elevated in PDAC and correlated with pancreatic cancer cell proliferation and migration [16, 17]. In 2013, Lai et al. showed that the ZBTB10-Sp1 pathway was involved in follicle stimulating hormone-induced COX2 expression in ovarian epithelial cancer cells [18]. Silva et al. also demonstrated that vorinostat, an HDAC inhibitor, promoted cell-cycle arrest, inhibited growth, and induced apoptosis and differentiation of acute myeloid leukemia and myelodysplastic syndrome cells by inhibiting DNA binding of Sp1 to the proximal promoter regions of some genes including COX2 [19]. In PDAC, another study revealed that celecoxib inhibited angiogenesis and metastasis via the suppression of Sp1 [14]. However, until now, the correlation between Sp1 and COX2 and their synergistic effect on the prognosis of PDAC patients remains unknown.

In this study, we evaluated the expression of Sp1 and COX2 by immunohistochemistry on tissue microarray slides and studied their prognostic value in the PDAC patients. We also investigated their correlation in PDAC cell lines. To our knowledge, this is the first study to analyze the correlation between Sp1 and COX2 and to investigate their combined prognostic value in PDAC.

## RESULTS

### Correlations between Sp1/COX2 and clinicopathological characteristics

Sp1 expression was significantly higher in PDAC cases with lymph node metastasis (*P*=0.02) than those without lymph node metastasis. Likewise, COX2 expression correlated with lymph node metastasis (*P*=0.03). However, no significant differences in Sp1 or COX2 expression were identified in patients of different age, sex, tumor stage, primary tumor location, lymphovascular invasion, nuclear grade, and clinical manifestation (Table 1).

**Table 1:** Correlation between Sp1, COX2 and clinicopathological features of PDAC patients

Factors	N	Sp1			COX2		
		Negative	Positive	P-value	Negative	Positive	P-value
Gender							
Male	56	21	35	0.56	17	39	0.83
Female	32	14	18		9	23	
Age							
≤60	41	17	24	0.76	11	30	0.60
>60	47	18	29		15	32	
T stage							
≤T2	74	31	43	0.35	20	54	0.38
T3	14	4	10		6	8	
N stage							
N0	52	26	26	0.02*	20	32	0.03*
N1	36	9	27		6	30	
Primary tumor location							
Body and Tail	30	15	15	0.16	8	22	0.67
Head and Neck	58	20	38		18	40	
Lymphovascular invasion							
No	50	22	28	0.35	15	35	0.92
Yes	38	13	25		11	27	
Nuclear grade							
≤II	73	31	42	0.26	19	54	0.20
>II	15	4	11		7	8	
Jaundice							
No	62	28	34	0.11	20	42	0.39
Yes	26	7	19		6	20	
Abdominal pain							
No	39	14	25	0.51	15	24	0.10
Yes	49	21	28		11	38	

Abbreviation: N, number; Sp1, specificity protein 1; COX2, cyclooxygenase-2. *P < 0.05 indicates a significant association among the variables.

### Correlation between Sp1 and COX2

**Figure 1:**
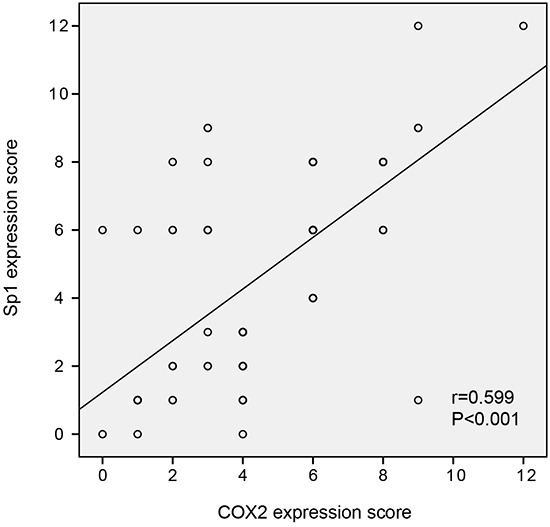
The correlation between Sp1 and COX2 expression in PDAC tissues Spearman's rank test demonstrated a significant correlation between Sp1 and COX2 (r=0.599; *P*<0.001).

To determine whether Sp1 expression is positively related to COX2 expression in PDAC samples, serial sections from the same PDAC tissue were scored for intracellular staining intensity of either Sp1 or COX2. Figure 1 depicts the co-distribution of Sp1 and COX2 in PDAC. Spearman's rank test demonstrated a significant correlation between Sp1 and COX2 (r=0.599; *P*<0.001).

### Correlation between Sp1/COX2 and the prognosis

**Figure 2:**
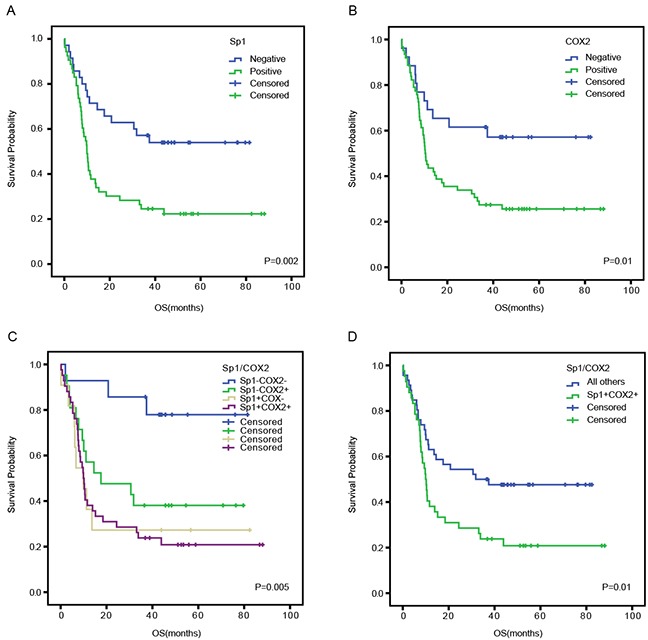
Overall survival curves based on Sp1 and COX2 expression in PDAC tissues The overall survival curves were based on Sp1 **A.**, COX2 **B.**, and the combination of Sp1 and COX2 (as polytomous variables and binary categorical variables, respectively) **C** and **D.**. All others: Sp1+COX2-, Sp1-COX2+, and Sp1-COX2-.

To investigate the prognostic value of Sp1 and COX2 in the survival of PDAC patients, a Kaplan–Meier analysis was conducted. We demonstrated that the median OS in Sp1-positive patients was 9.9 months which was significantly shorter than those of Sp1-negative patients (*P*=0.002, Figure 2A). Similarly, COX2-positive patients showed a worse prognosis (median OS: 10.4 months) than COX2-negative patients (median OS: 40.2 months) (*P*=0.01, Figure 2B). Intriguingly, patients with both Sp1- and COX2-positive expression exhibited worst OS than other conditions (*P*=0.005, Figure 2C). Likewise, when the latter three conditions were combined into a single variable named “all others”, a similar result was found (*P*=0.01, Figure 2D).

### Univariate and multivariate analysis

To evaluate the prognostic value of Sp1, COX2, and other clinicopathological characteristics, we performed a univariate analysis. N stage (*P*=0.006), nuclear grade (*P*=0.02), positive Sp1 or COX2 expression (*P*=0.003 and *P*=0.01, respectively), and combined positive expression of Sp1 and COX2 (*P*=0.01) were significantly associated with poor OS. According to the multivariate analysis, Sp1 expression (*P*=0.03; HR=4.48; 95% CI 1.14–17.62), COX2 expression (*P*=0.04; HR=3.84; 95% CI 1.08–13.71) and nuclear grade (*P*=0.009; HR=0.37; 95% CI 0.18–0.78) were independent prognostic factors for OS of PDAC patients. However, the N stage (*P*=0.07; HR=0.59; 95% CI 0.33–1.04) and combined positive expression of Sp1 and COX2 (*P*=0.16; HR=0.33; 95% CI 0.07–1.55) were not independently associated with OS (Table 2).

**Table 2:** Univariate and multivariate survival analysis of clinicopathological variables in PDAC patients

Factors	N	OS median(range)	Univariate analysis			Multivariate analysis		
			HR	95%CI	P-value	HR	95%CI	P-value
Gender								
Male	56	11.1(0.2-88.0)	0.58	0.33-1.03	0.07			
Female	32	36.7(0.1-76.4)	1					
Age								
>60	47	11.3(0.2-86.7)	1.05	0.63-1.77	0.84			
≤60	41	15.2(0.1-88.0)	1					
T stage								
T3	14	23.5(0.2-88.0)	1.03	0.50-2.09	0.95			
≤T2	74	12.6(0.1-86.7)	1					
N stage								
N0	52	33.5(0.2-88.0)	0.69	0.53-0.90	0.006*	0.59	0.33-1.04	0.07
N1	36	9.8(0.1-86.7)	1			1		
Primary tumor location								
Head and Neck	58	14.8(0.1-88.0)	0.83	0.49-1.43	0.51			
Body and Tail	30	10.5(1.3-79.6)	1					
Lymph vascular invasion								
Yes	38	10.6(0.2-82.4)	1.39	0.82-2.33	0.22			
No	50	18.0(0.1-88.0)	1					
Nuclear grade								
≤II	73	18.4(0.2-88.0)	0.68	0.49-0.93	0.02*	0.37	0.18-0.78	0.01*
>II	15	7.0(0.1-79.6)	1			1		
Jaundice								
No	62	14.2(0.2-82.4)	0.98	0.55-1.74	0.94			
Yes	26	11.5(0.1-88.0)	1					
Abdominal pain								
No	39	10.6(0.1-88.0)	0.71	0.42-1.19	0.19			
Yes	49	17.6(2.7-86.7)	1					
Sp1								
Positive	53	9.9(0.1-88.0)	2.42	1.35-4.33	0.003*	4.48	1.14-17.62	0.03*
Negative	35	37.4(0.2-81.5)	1			1		
COX2								
Positive	62	10.4(0.2-88.0)	2.28	1.18-4.42	0.01*	3.84	1.08-13.71	0.04*
Negative	26	40.2(0.1-82.4)	1			1		
Sp1/COX2								
Sp1+/COX2+	42	10.1(0.2-88.0)	1.95	1.15-3.30	0.01*	0.33	0.07-1.55	0.16
All others	46	34.2(0.1-82.4)	1			1		

Abbreviation: N, number; HR, hazard ratio; 95%CI, 95% confidence interval.

All others: Sp1+COX2-, Sp1-COX2+, and Sp1-COX2-.

### Sp1 promoted the expression of COX2 in PDAC cell lines

**Figure 3:**
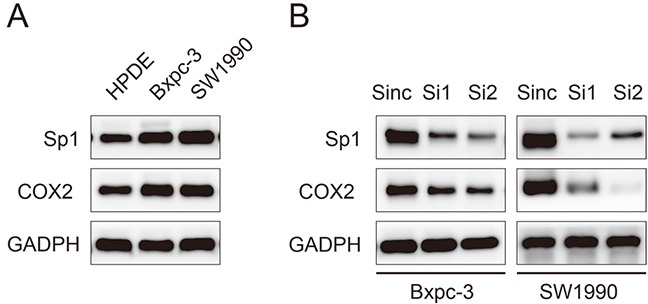
Sp1 promotes the expression of COX2 in human pancreatic cancer cell lines **A.** Western blot analysis of Sp1 and COX2 in HPDE, Bxpc-3, and SW1990 cells. **B.** Western blot analysis of Sp1 and COX2 in Sinc-, Si1-, and Si2-treated Bxpc-3 and SW1990 cells.

To confirm the association between Sp1 and COX2, we performed *in vitro* experiments. The expression of Sp1 and COX2 in HPDE pancreatic cells and two PDAC cell lines, Bxpc-3 and SW1990, were evaluated by western blot analysis. Higher expression of Sp1 and COX2 were observed in PDAC cells lines than in HPDE cells (Figure 3A). Sp1 was silenced by transient transfection of siRNAs in Bxpc-3 and SW1990 cell lines, which are characterized by high SP1 expression. The expression of Sp1 was successfully suppressed at 48 h after transfection and verified by western blot analysis. Accordingly, downregulated expression of COX2 was observed in Sp1 knockdown Bxpc-3 and SW1990 cell lines (Figure 3B).

## DISCUSSION

Our study corroborated previous studies that Sp1 and COX2 are upregulated in PDAC tissues and are inversely correlated with survival. Notably, we further found a novel correlation between the expression of Sp1 and COX2 in PDAC samples and validated this association in PDAC cell lines. To the best of our knowledge, this is the first study to investigate the correlation between Sp1 and COX2 and their prognostic value in PDAC.

As previous studies showed, Sp1 and COX2 play a critical role in the pathogenesis, aggressiveness, and angiogenesis of PDAC and their high expression usually indicates the presence of lymph node metastasis, advanced cancer stage, and reduced OS. We identified a positive correlation between Sp1, COX2, and the nodal stage in PDAC (*P*=0.02 and *P*=0.03, respectively). Node positivity is one of the typical indicators of pancreatic cancer metastasis. Jiang et al. clearly showed that every PDAC patient with SP1 overexpression had lymph node metastasis [12]. Wenjun Li et al. also reported that COX2 promoted PDAC cell migration through modifying the epithelial–mesenchymal transition process [17]. Collectively, it was suggested that the upregulated expression of COX2 by Sp1 in PDAC cells promotes tumor cell epithelial–mesenchymal transition and facilitates their migration and metastasis into lymphatic vessels [20]. This finding might assist in the decision to remove lymph nodes during surgery. However, neither Sp1 nor COX2 was correlated with tumor stage, lymphovascular invasion, and other clinicopathological factors, which may partially be explained by the small sample size of our study.

Several previous studies suggested that Sp1 may regulate the expression and function of COX2 in ovarian epithelial cancer and of acute myeloid leukemia [18, 19]. In this study, we also demonstrated a positive correlation between Sp1 and COX2 in PDAC samples (r=0.599; P<0.001) and further confirmed this observation in PDAC cell lines by transiently knocking down SP1. The potential clinical importance of this finding lies in the facts that current chemotherapeutic regimens do not provide PDAC patients with substantial survival benefit [21] and COX2 has been exploited in clinical trials of PDAC as a therapeutic target with inconsistent results and inevitable side effects [22–25]. Our study supported the idea that Sp1 regulates the expression of COX2 in PDAC cells. Such a finding may suggest a safe and efficacious mode of suppressing COX2 by targeting Sp1, which lacks the side effects related to COX2-inhibitory activity but has improved antineoplastic properties.

Both Sp1 and COX2 were found to be independent prognostic factors for PDAC in the present study, and their discriminative ability was almost the same. These findings strongly suggested the combination of Sp1 and COX2 may be of improved value in predicting PDAC patient survival. As expected, Kaplan–Meier analysis revealed that Sp1- and COX2-positive patients tended to have poorer prognosis than other conditions. However, the combined positive expression of Sp1 and COX2 was not demonstrated to be an independent prognostic factor in the Cox regression model, which may be explained by a defect in sample number and bias in the immunohistochemical evaluation.

In conclusion, our research demonstrated that Sp1 upregulates the expression of COX2 in PDAC, and that both are of significant prognostic value for PDAC patients. Further well-designed studies with larger sample sizes and new quantitative molecular techniques are required to evaluate this correlation.

## MATERIALS AND METHODS

### Cell culture

Pancreatic cancer cell lines Bxpc-3 and SW1990 were purchased from the Cell Bank of Chinese Academy of Science in Shanghai, China. Cells were cultured in RPMI-1640 (Gibco, Carlsbad, CA, USA) supplemented with 10% fetal bovine serum (Gibco) at 37°C in a humidified atmosphere of 95% air and 5% CO_2_, and subcultured by harvesting with trypsin-EDTA.

### RNA interference

RNA interference was conducted as previously described [26]. Small interfering RNAs (siRNAs) targeting Sp1 were chemically synthesized (Invitrogen, Shanghai, China). The siRNA oligonucleotides included Si1 (CAGCGUUUCUGCAGCUACCUUGACU) and Si2 (GACAGGUCAGUUGGCAGACUCUACA), while a vector (UUCUCCGAACGUGUCACGUdTdT) not targeting any annotated human genes was used as a negative control (Sinc). Transfection of siRNA duplexes into pancreatic cancer cells was carried out using Lipofectamine 2000 (Invitrogen) according to the manufacturer's instructions. At 48 h post-transfection, cells were harvested for western blot analysis.

### Western blot analysis

Cells were washed three times with cold PBS and lysed on ice in RIPA buffer containing PMSF protease inhibitor. The protein concentrations were determined using the BCA method (Beyotime Biotechnology, Haimen, China). A total of 30 μg of protein was separated by 10% SDS-PAGE and electro-blotted onto nitrocellulose membranes using a semi-dry blotting apparatus. After blocking in 3% bovine serum albumin, the membranes were incubated overnight at 4°C with primary antibodies against Sp1 and COX2 (Cell Signaling Technology, Beverly, MA, USA), and GAPDH (Santa Cruz Biotechnology, Santa Cruz, CA, USA) at 1μg/ml. The membranes were then incubated in the secondary antibodies for 1h at room temperature on a shaker. The protein bands were visualized by using a commercially available enhanced chemiluminescence kit (Thermo Scientific, Hudson, NH, USA). GAPDH was used as a loading control.

### Patients and specimens

From January 2009 to December 2012, 88 PDAC patients were enrolled at the Department of General Surgery, Shanghai General Hospital, Shanghai Jiao Tong University. Cancerous and adjacent normal tissue was collected during surgery, and histopathologically confirmed and staged according to the Union for International Cancer Control. Clinicopathological characteristics included age, sex, primary tumor location, clinical manifestation, and pathological stage (Table 3). Patients’ written informed consents and approval from the Ethics Committees of Shanghai General Hospital were obtained for the use of these clinical materials.

**Table 3:** Detailed characteristics of PDAC patients

Characteristics	Categories	Number
Gender	Male	56
	Female	32
Age median(range)		62(36-85)
T stage	T1	5
	T2	69
	T3	14
N stage	N0	52
	N1	36
TNM stage	IA	4
	IB	37
	IIA	11
	IIB	36
Primary tumor location	Head and Neck	58
	Body and Tail	30
Lymph vascular invasion	Yes	38
	No	50
Nuclear grade	I	1
	II	72
	III	15
Jaundice	Yes	26
	No	62
Abdominal pain	Yes	49
	No	39

### Tissue microarray construction

The microarray was made in collaboration with Shanghai Biochip, Shanghai, China. Representative tumor regions were defined as tumor areas containing more than 75% cancer cells without necrosis. Bleeding areas were neglected. Tissue cylinders (1.5 mm in diameter) were then punched from the defined regions of the block using a tissue microarray (Century, IL, CA, USA) and placed into recipient paraffin blocks. Two sets of three paraffin-embedded tissue microarray blocks were made, each containing 88 tumor tissue spots. Sections of the resulting tissue microarray blocks were transferred to glass slides.

### Immunohistochemistry

The standard IHC protocol used was described previously [27]. Briefly, the tissue microarrays were dewaxed and dehydrated in a xylene and alcohol bath solution. Endogenous peroxidase activity was then blocked with 0.3% hydrogen peroxide for 10 mins. After that, antigen retrieval was performed by setting the slides in 0.01 M citrate buffer (pH 6.0) at 98°C for 5 min using a microwave oven. The slides were cooled to room temperature and blocked by incubating with normal goat serum at room temperature for 1h, followed by incubation with the primarily antibodies (Cell Signaling Technology, Beverly, MA, USA) at 4°C overnight. The sections were then incubated with the HRP-labeled secondary antibody and were visualized by 3, 3′-diaminobenzidine staining.

### Evaluation of immunohistochemistry

**Figure 4:**
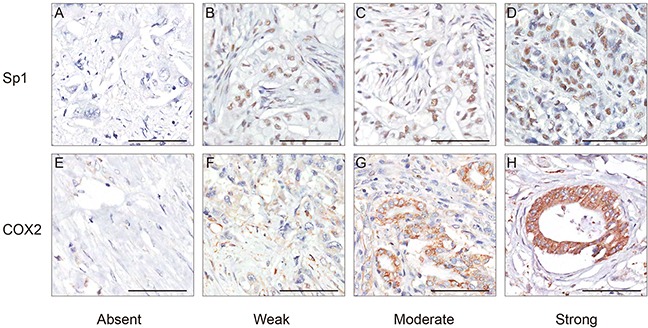
Representative immunohistochemical staining of Sp1 and COX2 in PDAC **A.** Absent staining of Sp1, score 0. **B.** Weak staining of Sp1, score 3. **C.** Moderate staining of Sp1, score 6. **D.** Strong staining of Sp1, score 12. **E.** Absent staining of COX2, score 0. **F.** Weak staining of COX2, score 1. **C.** Moderate staining of COX2, score 6. **D.** Strong staining of COX2, score 12. Bar: 100 μm.

**Figure 5:**
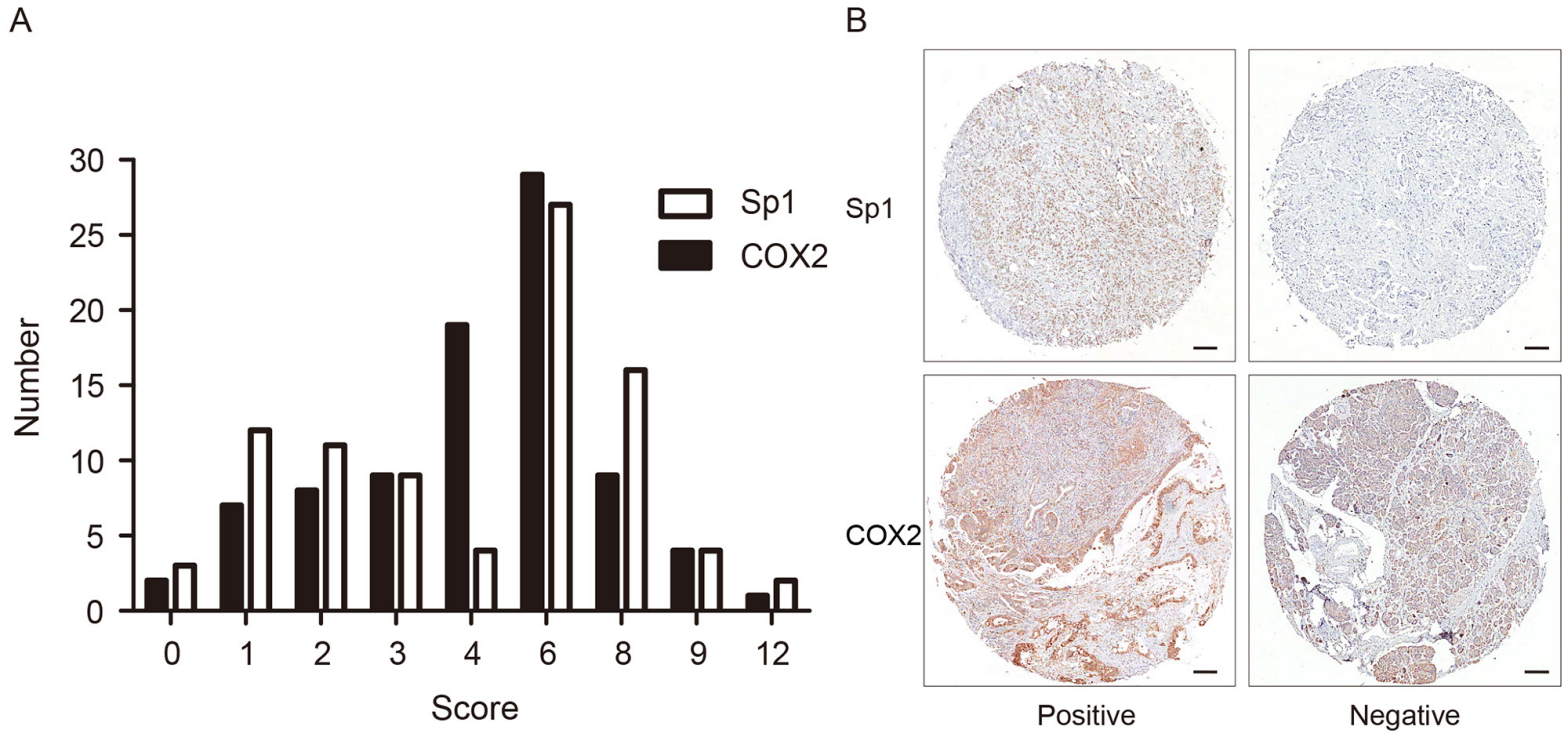
The distribution pattern and representative images of Sp1- and COX2- positive/negative expression **A.** The expression score and distribution pattern of Sp1 and COX2. **B.** Representative images of Sp1 and COX2 staining with positive/negative expression in PDAC tissues.Bar: 100 μm.

Sp1 and COX2 immunostaining signals were evaluated by two pathologists blinded to the clinical information. Tissues with brown cytoplasmic or nuclear staining for Sp1 and COX2 were considered positive. After scanning the stained sections at lower (×100) magnification, five areas with the greatest number of Sp1- or COX2-positive cells were selected. Then, these cells were counted and estimated (per mm^2^) at higher (×400) magnification. After the counting process, the percentage of Sp1- or COX2-positive cells was scored into the following five categories: 1 (< 25%); 2 (25% to 50%); 3 (50% to 75%); and 4 (> 75%). The staining intensity of positive cells was scored as 0 (absent); 1 (weak infiltration); 2 (moderate infiltration), and 3 (strong infiltration) (Figure 4). The final score was the product of the intensity and the percentage. The distribution pattern of Sp1 and COX2 expression score in PDAC was demonstrated in Figure 5A. For statistical analyses, these categories were further dichotomized into Sp1/COX2-negative (0-3) or -positive (4-12) (Figure 5B).

### Statistical analysis

All statistical analyses were performed using SPSS statistical software (version 21.0; SPSS Inc., Chicago, IL, USA). The relationships between Sp1 and COX2 protein expression and histological or clinical factors were investigated using the Pearson Chi-square test and Continuity Correction. The correlation between Sp1 and COX2 was evaluated with the Spearman's rank test. Kaplan–Meier analysis was used to demonstrate differences in overall survival (OS). The Cox regression model was used to evaluate the correlation between prognostic factors and OS. Factors correlated with the OS in the univariate analysis were further tested by multivariate analysis. The hazard ratio (HR) and corresponding 95% confidence interval (95% CI) were calculated for each factor. Results were considered statistically significant when *P*<0.05.
